# *N* ^4^,*N* ^6^-Dimethyl-*N* ^4^,*N* ^6^-diphenyl­pyrimidine-4,5,6-triamine

**DOI:** 10.1107/S1600536811046642

**Published:** 2011-11-09

**Authors:** Fuqiang Shi, Li-Hong Zhu, Li Mu, Long Zhang, Ya-Feng Li

**Affiliations:** a School of Chemical Engineering, Changchun University of Technology, Changchun 130012, People’s Republic of China; b School of Bioscience and Technology, Changchun University, Changchun 130012, People’s Republic of China

## Abstract

In the title compound, C_18_H_19_N_5_, the pyrimidine ring makes dihedral angles of 56.49 (9) and 70.88 (9)° with the phenyl rings. The dihedral angle between the two phenyl rings is 72.45 (9)°. No significant inter­molecular inter­actions are observed in the crystal structure.

## Related literature

For applications and the biological activity of pyrimidine triamines, see: Barillari *et al.* (2001 ▶); Itoh *et al.* (2004 ▶); Koppel & Robins (1958 ▶).
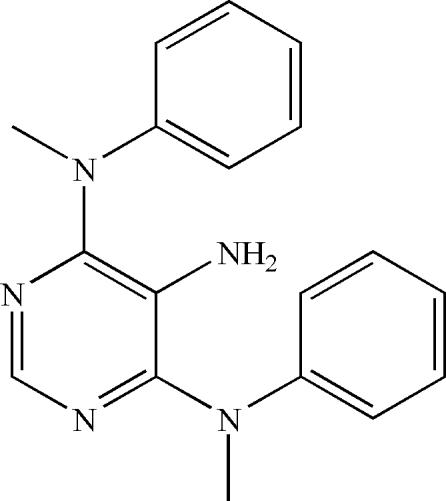


## Experimental

### 

#### Crystal data

C_18_H_19_N_5_
*M* *_r_* = 305.38
Orthorhombic, 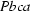

*a* = 8.8859 (18) Å
*b* = 14.360 (3) Å
*c* = 25.121 (5) Å
*V* = 3205.4 (11) Å^3^
*Z* = 8
Mo *K*α radiation
μ = 0.08 mm^−1^
*T* = 293 K
0.32 × 0.28 × 0.22 mm

#### Data collection

Rigaku R-AXIS RAPID diffractometer
Absorption correction: multi-scan (*ABSCOR*; Higashi, 1995 ▶) *T*_min_ = 0.975, *T*_max_ = 0.983
28152 measured reflections
3664 independent reflections
2119 reflections with *I* > 2σ(*I*)
*R*_int_ = 0.065

#### Refinement

*R*[*F* ^2^ > 2σ(*F* ^2^)] = 0.052
*wR*(*F* ^2^) = 0.137
*S* = 1.03
3664 reflections
210 parameters
H-atom parameters constrained
Δρ_max_ = 0.13 e Å^−3^
Δρ_min_ = −0.18 e Å^−3^

### 

Data collection: *PROCESS-AUTO* (Rigaku, 1998 ▶); cell refinement: *PROCESS-AUTO*; data reduction: *CrystalStructure* (Rigaku/MSC, 2002 ▶); program(s) used to solve structure: *SHELXS97* (Sheldrick, 2008 ▶); program(s) used to refine structure: *SHELXL97* (Sheldrick, 2008 ▶); molecular graphics: *DIAMOND* (Brandenburg, 2000 ▶); software used to prepare material for publication: *SHELXL97*.

## Supplementary Material

Crystal structure: contains datablock(s) I, global. DOI: 10.1107/S1600536811046642/is2800sup1.cif

Structure factors: contains datablock(s) I. DOI: 10.1107/S1600536811046642/is2800Isup2.hkl

Supplementary material file. DOI: 10.1107/S1600536811046642/is2800Isup3.cml

Additional supplementary materials:  crystallographic information; 3D view; checkCIF report

## Figures and Tables

**Fig. 1.**
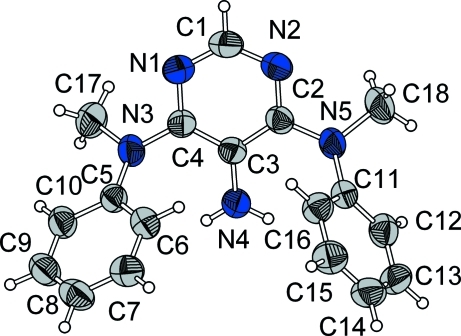
The molecular structure of the title compound, with the atom-labelling scheme. Displacement ellipsoid are shown at the 50% probability level.

## supplementary crystallographic information

### Comment

Pyrimidine triamines not only exhibit a wide range of biological activities (Barillari *et al.*, 2001), but also are important intermediate products (Koppel & Robins, 1958; Itoh *et al.*, 2004). Here, the crystal structure of *N*^4^,*N*^6^-dimethyl-*N*^4^,*N*^6^- diphenylpyrimidine-4,5,6-triamine is reported.

### Experimental

*N*^4^,*N*^6^-dimethyl-5-nitro-*N*^4^,*N*^6^ -diphenylpyrimidine-4, 6-diamine (502.5 mg, 1.5 mmol) was dissolved in a mixture of ethanol (16 mL) and water (4 mL). Then, iron powder (504 mg, 9 mmol) and NH_4_Cl (96.3 mg, 1.8 mmol) were added. The mixture was then stirred in reflux for 6 h, cooled to room temperature, and filtered through a pad of celite. The filtrate was concentrated in vacuo. The residue was extracted with EtOAc, and the organic extract was washed with saturated NaHCO_3_, water, and brine and dried over anhydrous MgSO_4_. It was then filtered and concentrated in vacuo to the crude product which was purified by flash chromatography (elution with 9% EtOAc in petroleum ether followed by 20% EtOAc in petroleum ether) to give *N*^4^,*N*^6^-dimethyl-*N*^4^,*N*^6^- diphenylpyrimidine-4,5,6-triamine (colorless solid, 310 mg, 67.8%, 88.6-90.6 °C).

### Refinement

All H atoms were located from difference Fourier maps and then were treated as riding, with C—H = 0.93–0.96 Å and N—H = 0.86 Å, and with *U*_iso_(H) = 1.2*U*_eq_(C or N) or 1.5*U*_eq_(methyl C).

### Crystal data

**Table:**

C_18_H_19_N_5_	*F*(000) = 1296
*M**_r_* = 305.38	*D*_x_ = 1.266 Mg m^−^^3^
Orthorhombic, *P**b**c**a*	Mo *K*α radiation, λ = 0.71073 Å
Hall symbol: -P 2ac 2ab	Cell parameters from 1000 reflections
*a* = 8.8859 (18) Å	θ = 3.2–27.5°
*b* = 14.360 (3) Å	µ = 0.08 mm^−^^1^
*c* = 25.121 (5) Å	*T* = 293 K
*V* = 3205.4 (11) Å^3^	Block, colorless
*Z* = 8	0.32 × 0.28 × 0.22 mm

### Data collection

**Table:**

Rigaku R-AXIS RAPID diffractometer	3664 independent reflections
Radiation source: fine-focus sealed tube	2119 reflections with *I* > 2σ(*I*)
graphite	*R*_int_ = 0.065
Detector resolution: 10.00 pixels mm^-1^	θ_max_ = 27.5°, θ_min_ = 3.2°
ω scans	*h* = −11→10
Absorption correction: multi-scan (*ABSCOR*; Higashi, 1995)	*k* = −18→18
*T*_min_ = 0.975, *T*_max_ = 0.983	*l* = −32→32
28152 measured reflections	

### Refinement

**Table:**

Refinement on *F*^2^	Primary atom site location: structure-invariant direct methods
Least-squares matrix: full	Secondary atom site location: difference Fourier map
*R*[*F*^2^ > 2σ(*F*^2^)] = 0.052	Hydrogen site location: inferred from neighbouring sites
*wR*(*F*^2^) = 0.137	H-atom parameters constrained
*S* = 1.03	*w* = 1/[σ^2^(*F*_o_^2^) + (0.0693*P*)^2^] where *P* = (*F*_o_^2^ + 2*F*_c_^2^)/3
3664 reflections	(Δ/σ)_max_ < 0.001
210 parameters	Δρ_max_ = 0.13 e Å^−^^3^
0 restraints	Δρ_min_ = −0.18 e Å^−^^3^

### Special details

**Table:**

Experimental. ^1^H NMR (CDCl_3_, 400 Hz), δ: 8.38 (s, 1H), 7.27 (t, J = 7.6Hz, 4H), 7.00(t, J = 7.2Hz, 2H), 6.90(d, J = 8.0Hz, 4H), 3.50 (s, 6H); 2.90 (s, 2H). ^13^C NMR (CDCl_3_, 100 Hz), δ: 151.1, 148.0, 145.7, 129.3, 122.8, 122.7, 120.0, 39.7. ES-MS: 336.1 [(M + H^+^)].
Geometry. All e.s.d.'s (except the e.s.d. in the dihedral angle between two l.s. planes) are estimated using the full covariance matrix. The cell e.s.d.'s are taken into account individually in the estimation of e.s.d.'s in distances, angles and torsion angles; correlations between e.s.d.'s in cell parameters are only used when they are defined by crystal symmetry. An approximate (isotropic) treatment of cell e.s.d.'s is used for estimating e.s.d.'s involving l.s. planes.
Refinement. Refinement of *F*^2^ against ALL reflections. The weighted *R*-factor *wR* and goodness of fit *S* are based on *F*^2^, conventional *R*-factors *R* are based on *F*, with *F* set to zero for negative *F*^2^. The threshold expression of *F*^2^ > σ(*F*^2^) is used only for calculating *R*-factors(gt) *etc*. and is not relevant to the choice of reflections for refinement. *R*-factors based on *F*^2^ are statistically about twice as large as those based on *F*, and *R*- factors based on ALL data will be even larger.

### Fractional atomic coordinates and isotropic or equivalent isotropic displacement parameters (Å^2^)

**Table:**

	*x*	*y*	*z*	*U*_iso_*/*U*_eq_	
C1	−0.0279 (3)	0.46280 (13)	0.41196 (8)	0.0660 (6)	
H1	−0.0833	0.5128	0.4249	0.079*	
C2	0.0751 (2)	0.32120 (11)	0.42746 (6)	0.0483 (4)	
C3	0.15006 (19)	0.32587 (11)	0.37827 (6)	0.0460 (4)	
C4	0.1152 (2)	0.40201 (11)	0.34631 (6)	0.0484 (4)	
C5	0.1622 (2)	0.34917 (11)	0.25472 (6)	0.0480 (4)	
C6	0.0766 (2)	0.26882 (12)	0.26084 (7)	0.0563 (5)	
H6	0.0287	0.2573	0.2931	0.068*	
C7	0.0620 (2)	0.20590 (13)	0.21954 (8)	0.0626 (5)	
H7	0.0059	0.1519	0.2245	0.075*	
C8	0.1296 (2)	0.22228 (16)	0.17087 (8)	0.0689 (6)	
H8	0.1205	0.1795	0.1433	0.083*	
C9	0.2099 (2)	0.30237 (15)	0.16412 (8)	0.0679 (6)	
H9	0.2534	0.3148	0.1312	0.082*	
C10	0.2278 (2)	0.36518 (14)	0.20496 (7)	0.0586 (5)	
H10	0.2842	0.4189	0.1994	0.070*	
C11	0.0814 (2)	0.15421 (10)	0.44409 (6)	0.0478 (4)	
C12	0.1647 (2)	0.08195 (13)	0.46579 (7)	0.0612 (5)	
H12	0.2369	0.0943	0.4917	0.073*	
C13	0.1404 (3)	−0.00819 (13)	0.44895 (9)	0.0713 (6)	
H13	0.1949	−0.0566	0.4642	0.086*	
C14	0.0367 (3)	−0.02727 (13)	0.40991 (9)	0.0732 (6)	
H14	0.0223	−0.0882	0.3983	0.088*	
C15	−0.0447 (3)	0.04322 (13)	0.38829 (8)	0.0688 (6)	
H15	−0.1150	0.0305	0.3619	0.083*	
C16	−0.0238 (2)	0.13385 (12)	0.40531 (7)	0.0570 (5)	
H16	−0.0810	0.1815	0.3905	0.068*	
C17	0.2467 (3)	0.50505 (12)	0.28371 (9)	0.0743 (6)	
H17A	0.3482	0.4977	0.2713	0.111*	
H17B	0.2464	0.5430	0.3152	0.111*	
H17C	0.1874	0.5345	0.2566	0.111*	
C18	0.0744 (4)	0.26304 (14)	0.51905 (7)	0.0931 (9)	
H18A	0.1106	0.3236	0.5290	0.140*	
H18B	0.1257	0.2163	0.5395	0.140*	
H18C	−0.0318	0.2593	0.5258	0.140*	
N1	0.02716 (19)	0.47211 (9)	0.36360 (6)	0.0593 (4)	
N2	−0.01344 (19)	0.39000 (10)	0.44460 (6)	0.0602 (4)	
N3	0.18291 (19)	0.41347 (9)	0.29607 (6)	0.0578 (4)	
N4	0.25718 (18)	0.26103 (9)	0.36391 (6)	0.0583 (4)	
H4A	0.3049	0.2669	0.3343	0.070*	
H4B	0.2762	0.2147	0.3845	0.070*	
N5	0.1029 (2)	0.24762 (9)	0.46223 (5)	0.0568 (4)	

### Atomic displacement parameters (Å^2^)

**Table:**

	*U*^11^	*U*^22^	*U*^33^	*U*^12^	*U*^13^	*U*^23^
C1	0.0737 (15)	0.0553 (10)	0.0688 (13)	0.0106 (10)	0.0113 (11)	−0.0102 (9)
C2	0.0550 (11)	0.0480 (9)	0.0419 (9)	−0.0040 (8)	−0.0006 (8)	−0.0048 (7)
C3	0.0475 (10)	0.0451 (8)	0.0454 (9)	−0.0010 (8)	0.0001 (8)	−0.0040 (7)
C4	0.0528 (11)	0.0448 (8)	0.0475 (10)	−0.0015 (8)	−0.0008 (8)	−0.0016 (7)
C5	0.0473 (10)	0.0530 (9)	0.0437 (9)	0.0073 (8)	−0.0007 (7)	0.0038 (7)
C6	0.0542 (11)	0.0653 (11)	0.0493 (10)	−0.0004 (9)	0.0027 (9)	0.0004 (8)
C7	0.0603 (13)	0.0644 (11)	0.0631 (12)	0.0040 (10)	−0.0090 (10)	−0.0071 (9)
C8	0.0674 (14)	0.0877 (14)	0.0517 (12)	0.0182 (12)	−0.0070 (10)	−0.0156 (10)
C9	0.0669 (14)	0.0877 (14)	0.0492 (11)	0.0198 (12)	0.0063 (10)	−0.0004 (10)
C10	0.0547 (12)	0.0690 (11)	0.0522 (11)	0.0099 (9)	0.0064 (9)	0.0093 (9)
C11	0.0520 (10)	0.0490 (9)	0.0425 (9)	−0.0001 (8)	0.0037 (8)	0.0017 (7)
C12	0.0571 (12)	0.0726 (12)	0.0540 (11)	0.0066 (10)	0.0002 (9)	0.0098 (9)
C13	0.0785 (16)	0.0578 (11)	0.0776 (14)	0.0206 (11)	0.0180 (12)	0.0118 (10)
C14	0.0905 (18)	0.0519 (11)	0.0770 (15)	−0.0024 (11)	0.0167 (13)	−0.0077 (10)
C15	0.0737 (15)	0.0623 (12)	0.0703 (13)	−0.0102 (11)	−0.0048 (11)	−0.0086 (10)
C16	0.0578 (12)	0.0547 (10)	0.0585 (11)	0.0006 (9)	−0.0063 (9)	0.0003 (8)
C17	0.0932 (17)	0.0561 (11)	0.0734 (14)	−0.0147 (11)	0.0105 (12)	0.0092 (9)
C18	0.164 (3)	0.0758 (13)	0.0391 (11)	−0.0086 (15)	0.0004 (13)	−0.0048 (9)
N1	0.0656 (11)	0.0511 (8)	0.0611 (10)	0.0072 (8)	0.0001 (8)	−0.0028 (7)
N2	0.0707 (11)	0.0551 (8)	0.0548 (9)	0.0016 (8)	0.0109 (8)	−0.0073 (7)
N3	0.0740 (12)	0.0528 (8)	0.0466 (8)	−0.0090 (8)	0.0073 (7)	0.0052 (6)
N4	0.0607 (10)	0.0602 (9)	0.0542 (9)	0.0132 (8)	0.0116 (8)	0.0062 (7)
N5	0.0807 (12)	0.0530 (8)	0.0366 (8)	−0.0063 (8)	−0.0048 (7)	−0.0014 (6)

### Geometric parameters (Å, °)

**Table:**

C1—N1	1.316 (2)	C11—C16	1.381 (2)
C1—N2	1.335 (2)	C11—C12	1.386 (2)
C1—H1	0.9300	C11—N5	1.429 (2)
C2—N2	1.334 (2)	C12—C13	1.379 (3)
C2—N5	1.393 (2)	C12—H12	0.9300
C2—C3	1.406 (2)	C13—C14	1.374 (3)
C3—N4	1.379 (2)	C13—H13	0.9300
C3—C4	1.391 (2)	C14—C15	1.357 (3)
C4—N1	1.347 (2)	C14—H14	0.9300
C4—N3	1.408 (2)	C15—C16	1.383 (2)
C5—C6	1.391 (2)	C15—H15	0.9300
C5—C10	1.398 (2)	C16—H16	0.9300
C5—N3	1.402 (2)	C17—N3	1.465 (2)
C6—C7	1.382 (2)	C17—H17A	0.9600
C6—H6	0.9300	C17—H17B	0.9600
C7—C8	1.382 (3)	C17—H17C	0.9600
C7—H7	0.9300	C18—N5	1.466 (2)
C8—C9	1.364 (3)	C18—H18A	0.9600
C8—H8	0.9300	C18—H18B	0.9600
C9—C10	1.375 (3)	C18—H18C	0.9600
C9—H9	0.9300	N4—H4A	0.8600
C10—H10	0.9300	N4—H4B	0.8600
			
N1—C1—N2	127.64 (17)	C14—C13—C12	120.75 (19)
N1—C1—H1	116.2	C14—C13—H13	119.6
N2—C1—H1	116.2	C12—C13—H13	119.6
N2—C2—N5	117.64 (15)	C15—C14—C13	119.64 (19)
N2—C2—C3	121.86 (15)	C15—C14—H14	120.2
N5—C2—C3	120.22 (15)	C13—C14—H14	120.2
N4—C3—C4	122.23 (15)	C14—C15—C16	120.4 (2)
N4—C3—C2	121.66 (15)	C14—C15—H15	119.8
C4—C3—C2	116.04 (15)	C16—C15—H15	119.8
N1—C4—C3	122.05 (15)	C11—C16—C15	120.57 (17)
N1—C4—N3	116.75 (14)	C11—C16—H16	119.7
C3—C4—N3	120.91 (16)	C15—C16—H16	119.7
C6—C5—C10	117.61 (16)	N3—C17—H17A	109.5
C6—C5—N3	122.42 (15)	N3—C17—H17B	109.5
C10—C5—N3	119.97 (16)	H17A—C17—H17B	109.5
C7—C6—C5	120.74 (17)	N3—C17—H17C	109.5
C7—C6—H6	119.6	H17A—C17—H17C	109.5
C5—C6—H6	119.6	H17B—C17—H17C	109.5
C6—C7—C8	120.78 (19)	N5—C18—H18A	109.5
C6—C7—H7	119.6	N5—C18—H18B	109.5
C8—C7—H7	119.6	H18A—C18—H18B	109.5
C9—C8—C7	118.74 (18)	N5—C18—H18C	109.5
C9—C8—H8	120.6	H18A—C18—H18C	109.5
C7—C8—H8	120.6	H18B—C18—H18C	109.5
C8—C9—C10	121.40 (19)	C1—N1—C4	115.94 (15)
C8—C9—H9	119.3	C2—N2—C1	116.00 (16)
C10—C9—H9	119.3	C5—N3—C4	122.09 (14)
C9—C10—C5	120.70 (19)	C5—N3—C17	118.99 (15)
C9—C10—H10	119.7	C4—N3—C17	117.41 (14)
C5—C10—H10	119.7	C3—N4—H4A	120.0
C16—C11—C12	118.70 (16)	C3—N4—H4B	120.0
C16—C11—N5	120.91 (15)	H4A—N4—H4B	120.0
C12—C11—N5	120.38 (16)	C2—N5—C11	119.23 (13)
C13—C12—C11	119.90 (19)	C2—N5—C18	117.72 (15)
C13—C12—H12	120.0	C11—N5—C18	115.39 (14)
C11—C12—H12	120.0		
